# Lifetime Multiple Mild Traumatic Brain Injuries Are Associated with Cognitive and Mood Symptoms in Young Healthy College Students

**DOI:** 10.3389/fneur.2016.00188

**Published:** 2016-10-31

**Authors:** Kyle C. Vynorius, Alyssa M. Paquin, Daniel R. Seichepine

**Affiliations:** ^1^Neuropsychology Laboratory, University of New Hampshire Manchester, Manchester, NH, USA

**Keywords:** mild traumatic brain injury, neuropsychology, executive function, cognition

## Abstract

**Background/objectives:**

Repetitive mild traumatic brain injury (mTBI, also known as concussion) has been associated with a range of long-term mood and cognitive deficits, including executive dysfunction. Previous research in athletes suggests that cognitive and mood problems are associated with a history of repetitive mTBI. However, to date, no studies have examined the impact of a lifetime accumulation of repetitive mTBIs on cognition, particularly executive functioning, and mood in a sample of young adults who were not athletes. Therefore, the present study looked at potential effects of repetitive mTBIs on self-reported cognitive complaints, executive functioning, and mood in young adults.

**Methods:**

Eighty-four total students responded, and 26 of those were excluded from analyses due to reporting only 1 mTBI. The final sample consisted of 58 healthy young adults (mean age = 22.84, STD = 4.88) who completed the Cognitive Complaint Index (CCI), the Behavior Rating Inventory of Executive Function, adult version (BRIEF-A), and the Beck Depression Inventory, second edition (BDI-II). Twenty-nine participants denied having an mTBI history, and 29 reported 2 or more lifetime mTBIs (range 2–7).

**Results:**

Young otherwise healthy adults with a lifetime history of repetitive mTBI compared to those that reported no history of mTBI reported more change in cognitive functioning over the past 5 years, worse executive functioning, and more symptoms of depression. As the number of lifetime mTBIs increased, scores on the CCI, BRIEF-A, and BDI-II also increased, indicating worse functioning.

**Conclusion:**

These findings suggest that a lifetime accumulation of two or more mTBIs as compared to a history of no reported mTBIs may result in worse cognitive functioning and symptoms of depression in young adults.

## Introduction

Traumatic brain injury (TBI) is a significant public heath problem in the United States. Approximately 2.5 million Americans seek medical services annually for the treatment of mild to severe TBI (1). Given that mild TBIs (mTBIs) are thought to be a “silent epidemic” that often go unrecognized and underreported, there is no consensus on how many of these injuries occur annually (2). Within the sports literature alone, it is estimated that between 1.6 and 3.8 million mTBIs occur annually; however, this excludes injuries due to other causes (falls, accidents, assaults, etc.) and likely underestimates the total number of injuries that occur (3). Within the military, mTBIs have been described as the “signature” injury of the recent wars in Iraq and Afghanistan and have affected more than 300,000 service members since 2000 (4).

Mild traumatic brain injury occurs when a force to the head or body results in the transient disruption of brain functioning for less than 30 minutes, alteration of mental state for less than 24 h, and posttraumatic amnesia for less than 1 day (5, 6). These injuries can cause a wide variety of acute (lasting 1 month or less) and chronic (lasting several months or more) neurological symptoms. These include changes in cognition, emotion, and sensation as well as headaches, nausea, fatigue, and sleep disturbances (7).

Traditionally, mTBI has been viewed exclusively as a temporary change in brain functioning. However, recent neuroimaging evidence, using diffusion tensor imaging (DTI), indicates that these injuries may also result in structural changes to white matter pathways in the brain (8). DTI indicates that neurocognitive deficits after mTBI may lead to impaired executive functioning due to axonal injury in the frontal lobe (9). Changes in these pathways may contribute to the chronic symptoms of mTBI such as poor memory, poor concentration, and sadness (8, 10). For example, retired former professional football players with multiple mTBIs report more cognitive and behavioral symptoms and have higher axial diffusivity on DTI when compared to healthy controls, suggesting that an accumulation of mTBIs over a lifetime is associated with both white matter changes and overt symptoms (11).

In addition to white matter changes, mTBI is also associated with more severe long-term structural changes. For example, extensive atrophy and tauopathy have been observed post-mortem in former male athletes and military personal with a history of repetitive mTBI (12, 13). This neurodegenerative disease, termed chronic traumatic encephalopathy (CTE), is thought to be progressive and stem from exposure to repetitive brain injury.

In the majority of cases, the symptoms of an mTBI resolve within a couple of months. Repetitive exposure to mTBIs, such as those experienced by contact-sports athletes (14–16) and military personnel, has been linked to long-term problems with executive functioning. Executive functioning is the management, regulation, and execution of cognitive processes such as working memory, task switching, reasoning, and problem solving. Previous research suggests problems with executive functioning, within individuals with repetitive mTBIs, emerge by the fifth decade of life, even though the brain injuries occurred decades earlier (14). To date, no studies have examined the impact of lifetime accumulation of repetitive mTBIs on cognition, particularly executive functioning, and mood in a sample of young adults who were not elite athletes. Therefore, the present study examined the influence of repetitive mTBIs acquired over a lifetime on cognitive complaint, self-reported executive functioning, and symptoms of depression.

## Materials and Methods

### Participants

Eighty-four total students responded, and 26 of those were excluded from analyses due to reporting only 1 mTBI. The final sample consisted of 58 young healthy college students (44 women and 14 men), who were grouped by number of self-reported lifetime mTBIs accordingly: without mTBI (no-mTBI; 25 women, 4 men) or repetitive mTBI (r-mTBI; 19 women, 10 men). Groups were similar in age [*t*(56) = 1.05, *p* = 0.298], education [*t*(56) = 1.18, *p* = 0.241], and male-to-female ratio (*x*^2^ =3.39, *p* = 0.066). Participants were recruited by flyers posted on campus, which contained a link to the anonymous online survey. Participants were compensated for their time. Characteristics of the no-mTBI and r-mTBI groups are listed in Table 1. All participants provided informed consent for the protocol approved by the University of New Hampshire Institutional Review Board.

**Table 1:** **Participant characteristics**.

	No mTBI (*n* = 29)	Repetitive mTBI (*n* = 29)	*p*-Value
Age	22.17 (4.83)	23.51 (4.92)	0.298
Female:male	25:4	19:10	0.066
Education (years)	14.41 (1.21)	14.90 (1.57)	0.194
Total lifetime number of mTBIs	N/A	3.0 (2–7)	N/A

*Values represent means and SDs, except number of mild traumatic brain injuries which is reported in median and range*.

### Measure and Procedure

Participants completed an anonymous, self-report online survey assessing demographic characteristics, number of mTBIs and respective time(s)/causes of occurrence, and the following well-established and standardized measures of cognition and executive functioning, and depression.

To assess the number of self-reported lifetime mTBIs, participants were provided with a definition of concussion consistent with current guidelines from the American Academy of Neurology, which has been used in several recent publications (14, 17). This definition clarifies what constitutes an mTBI and provides examples of common symptoms. The following mTBI definition was provided:

There is a misconception that concussions (also known as “mild traumatic brain injury”) only happen when you lose consciousness after being hit on the head or when the symptoms last for a long time. However, a concussion occurs anytime you have an impact to the head that causes neurological symptoms for any amount of time (e.g., seconds or longer). Common symptoms of concussion include: sensitivity to light or noise, headache, dizziness, balance problems, nausea, vomiting, trouble sleeping, fatigue, confusion, difficulty remembering, difficulty concentrating, or loss of consciousness.

#### Characteristics of Most Severe Traumatic Brain Injury

None of the participants reported an injury severe enough to result in a skull fracture. Twenty-one of the 29 participants with an mTBI history reported receiving medical care for their injury (72.4%), but only 6 reported receiving a CT or MRI scan (20.7%). Fourteen of the 29 participants (48.3%) reported experiencing loss of consciousness. Participants were not asked about the length of unconsciousness. Eight participants reported being prescribed medication and three reported receiving occupational therapy.

#### Beck Depression Inventory, Second Edition

The Beck Depression Inventory, second edition (BDI-II) is a 21-item self-report inventory used to measure symptoms of depression over the last 2 weeks (18). This measure assesses sadness, pessimism, irritability, concentration, and energy. Scores on each of the items range from 0 to 3, where 0 indicates no symptoms and 3 indicates the most number of symptoms. A score of 0 indicates no symptoms; 1 indicates some symptoms; 2 indicates more symptoms; and 3 indicates the most symptoms. Higher scores indicate more symptoms of depression. This measure yields an overall score.

#### Cognitive Complaint Index

The Cognitive Complaint Index (CCI) is a 20-item self-report inventory of current cognitive function compared to 5 years ago (19). Scores on each of the items range from 1 to 5, where 1 indicates no cognitive change and 5 indicates severe cognitive change. A score of 1 indicates normal ability (no changes); 2 indicates slight/occasional problems (minimal change); 3 indicates mild problems (some change); 4 indicates moderate problems (clearly noticeable change); and 5 indicates severe problems (much worse). Higher scores indicate more negative self-reported change. This measure yields an overall score.

#### Behavior Rating Inventory of Executive Function, Adult Version

The Behavior Rating Inventory of Executive Function, adult version (BRIEF-A) is a 75-item self-report inventory of executive function over the last 30 days (20). Participants indicate how problematic a behavior has been on the following 3-point scale: never (1 point); sometimes (2 points); or often (3 points). Higher scores indicate more executive dysfunction. This measure yields an overall score, the Global Executive Composite (GEC), which is the sum of the following two subscales: Behavior Regulation Index (BRI) and Metacognition Index (MI). The BRI measures the ability to maintain control over behavior and emotional responses. The MI measures the ability to solve problems through planning, organization, and sustained working memory.

## Results

Histograms for each of the dependent variables demonstrated that the data had a non-normal distribution; therefore, non-parametric statistics were used. Mann–Whitney *U* tests revealed that participants with r-mTBI compared to those that reported no-mTBI reported more symptoms of depression (*U* = 246.00, *p* = 0.006), more change in cognitive functioning over the past 5 years (*U* = 241, *p* = 0.005), and more executive functioning problems (*U* = 269.00, *p* = 0.018). Given that significant group differences were observed on the overall scale on the BRIEF-A (GEC), follow-up tests were performed on the two subscales. Significant group differences were observed on both of these subscales: BRI (*U* = 274.50, *p* = 0.023) and the MI (*U* = 278.00, *p* = 0.027). In both cases, the r-mTBI group reported more symptoms than that of the no-mTBI group. Scores on the CCI, Beck Depression Inventory, second edition (BDI-II), and BRIEF-A are listed in Table 2.

**Table 2:** **Cognitive Complaint Index, BDI-II, and BRIEF-A scores of individuals with no mTBI compared to individuals with repetitive mTBI (two or more)**.

	No mTBI (*n* = 29)	Repetitive mTBI (*n* = 29)	*p*-Value
Beck Depression Inventory, second edition	23.48 (681.00)	35.52 (1030.00)	0.006
Cognitive Complaint Index^a^	23.31 (676.00)	35.69 (1035.00)	0.005
**BRIEF-A Index Scores**			
GEC^a^	24.28 (704.00)	34.72 (1007.00)	0.018
BRI^a^	24.47 (709.50)	34.53 (1001.50)	0.023
MI^a^	24.59 (713.00)	34.41 (998.00)	0.027

*GEC, Global Executive Composite; BRI, Behavioral Regulation Index; MI, Metacognition Index; mean ranks and sum of ranks reported*.

*^a^Statistically significant at an alpha level of 0.05*.

**Figure 1:**
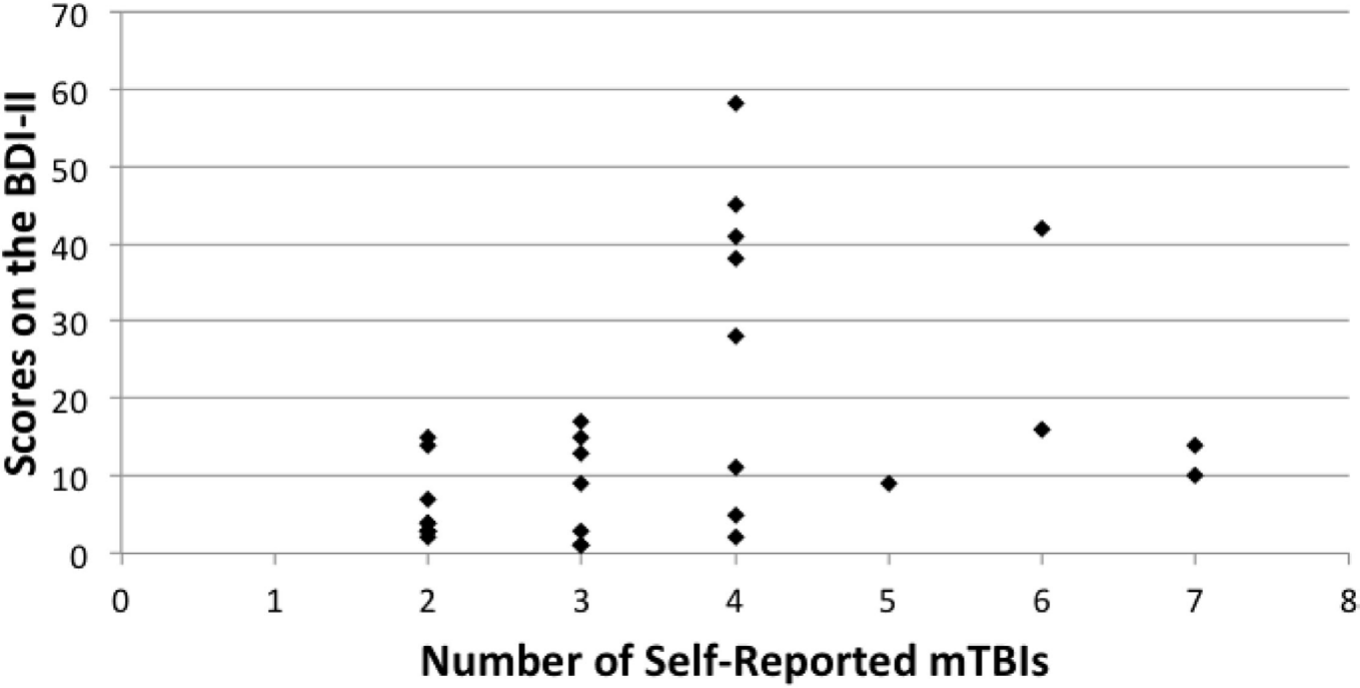
**Correlation between scores on the BDI-II and number of self-reported mTBIs**.

**Figure 2:**
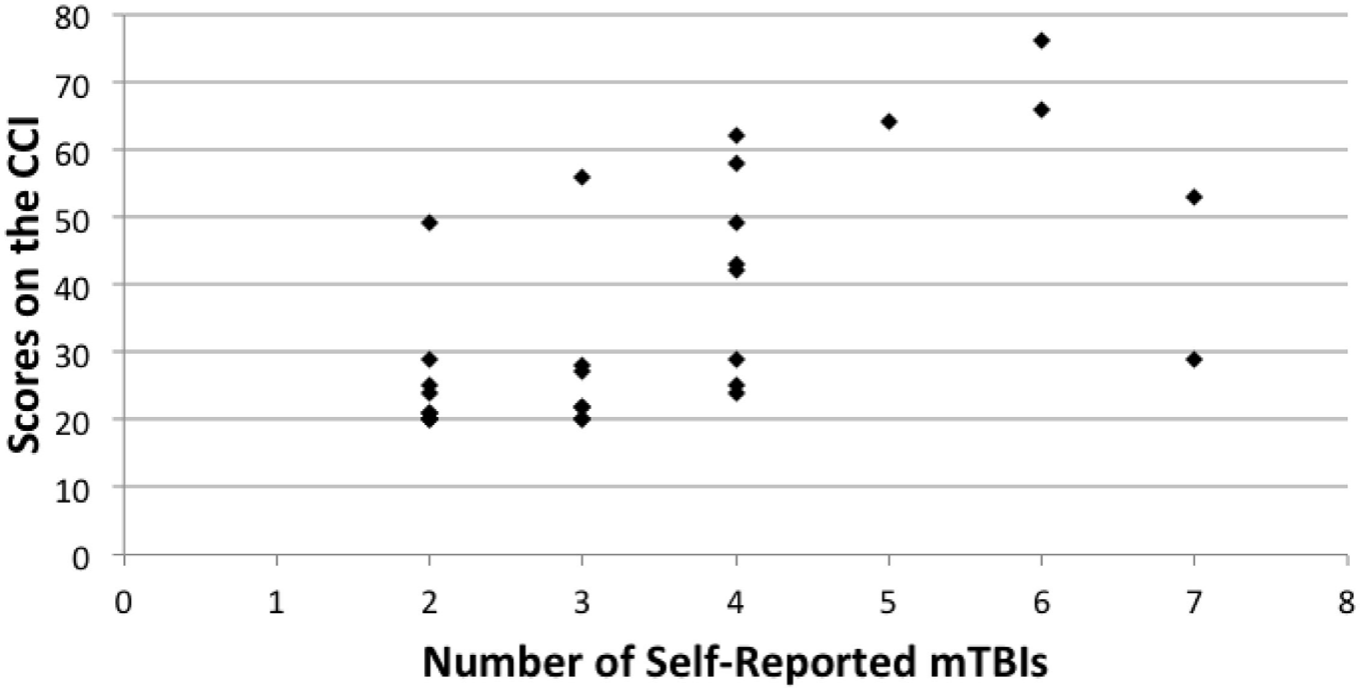
**Correlation between scores on the CCI and number of self-reported mTBIs**.

**Figure 3:**
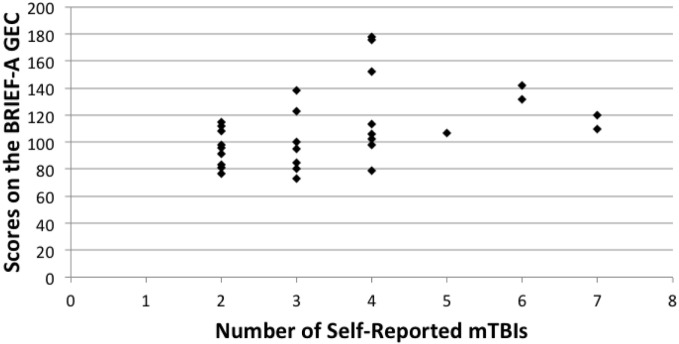
**Correlation between scores on the BRIEF-GEC and number of self-reported mTBIs**.

Spearman correlation coefficients were used to compare overall scores on the BDI-II, CCI, and BRIEF-A. Number of r-mTBIs correlated positively with the CCI (rho = 0.513, *p* < 0.000), BDI-II (rho = 0.477, *p* < 0.000), and the overall score on the BRIEF-A (rho = 0.472, *p* = 0.010). A significant correlation was also observed on the BRIEF-A MI subscale (rho = 0.460, *p* = 0.012), but not on the BRI subscale (rho = 0.346, *p* = 0.066). In all cases, as the number of mTBIs increased, the number of symptoms associated with each measurement increased as well. Figures 1–3 show scatter plots for the relation between self-reported mTBIs and overall scores on the BDI-II, CCI, and BRIEF-A.

## Discussion

Young otherwise healthy undergraduate students with a history of repetitive mTBI compared to those that reported no history of mTBI reported more change in cognitive functioning over the past 5 years, more executive functioning problems over the past 30 days, and more symptoms of depression over the past 2 weeks. These findings suggest that a lifetime accumulation of two or more concussions as compared to a history of no reported concussions may result in worsening symptoms of cognitive function, and increased executive dysfunction and depression. In general, the most common cognitive complaints included remembering names and faces of new people, remembering without notes or reminders, remembering things compared to those within the individuals’ age group, and the perception that people who know the individual would say that their memory is worse. In regard to symptoms of depression, the most common symptoms reported were changes in sleep patterns and appetite, as well as tiredness or fatigue. Interestingly in athletes, fatigue in the acute stage has previously been identified as risk factors for long-term cognitive and behavioral symptoms (21). In regard to executive function, the weaknesses were spread between both metacognitive and behavioral functions.

Consistent with another report in college and professional football players (14), the present study found significant correlations between number of self-reported mBTIs and self-reported rating of cognitive change, executive functioning, and symptoms of depression. Both studies found that as the number of self-reported mTBIs increases, so does self-reported symptoms. Given the recent publicity surrounding CTE in football players, the findings from the former study may have been influenced by only symptomatic individuals volunteering for the study. Given the population in the present study, the effects of this media coverage is likely lessened and therefore provides more evidence indicating support for a relation between number of mTBIs and changes in mood and cognition.

Overall, the present findings are consistent with a growing body of evidence indicating that exposure to mTBIs is associated with long-term problem in mood and cognition, particularly in athletes and military personnel. Within the military, TBI has been linked to self-reported physical and cognitive problems in veterans from the recent wars in Iraq and Afghanistan (22, 23). As these veterans continue to return home and reintegrate into civilian life, these findings suggest they may experience subtle symptoms associated with these injuries.

Though we believe that this study has many strengths, there are also several limitations that should be acknowledged. Given that the sample size was relatively small (58 undergraduate students) and that the participants were from a single urban New England institution, the results may not generalize to all undergraduate students. Given the recent media attention on the long-term effects of mTBIs on mood, cognition, and behavior, it is possible that participants believed there should be a relation between these factors and therefore responded accordingly. Additionally, participants may have had a tendency toward a “yea saying” bias and future studies should include an innocuous questionnaire about an unrelated topic (e.g., food preference) to address this potential bias. Variables such as socioeconomic status, learning disabilities, and average amount of sleep were not specifically addressed in the survey for this study, which may have contributed to the results collected between the BDI-II, CCI, and BRIEF-A.

This studied relied on self-report data, which may or may not correlate with objective neuropsychological testing. Therefore, a follow-up study using neuropsychological testing may be helpful in better understanding the relation between lifetime mTBIs and cognition. Finally, participants’ reporting of number of lifetime mTBIs may be inaccurate. It should be highlighted that in the present study, participants were provided a definition of mTBI that has been used in previous studies to assess the number of lifetime injuries (14, 24) and has been shown to affect the number of injuries reported overall (17). Though this increase may be attributed to demand characteristics, it is important to highlight that a previous study found that providing this definition did not change the number of self-reported mTBIs for 25% of the sample (17). Nonetheless, future studies may consider using a structured clinical interview, such as the Ohio State University TBI Identification Method – Interview Form.

In sum, young otherwise healthy undergraduate students with a history of repetitive mTBI reported more cognitive problems and symptoms of depression when compared to individuals who have not experienced an mTBI. These findings suggest that a lifetime accumulation of mTBIs may result in subtle changes in mood and cognitive symptoms even in highly functioning individuals.

## Author Contributions

All authors contributed significantly to the study design, data collection, analysis, and interpretation as well as to prepare the manuscript.

## Conflict of Interest Statement

The authors declare that the research was conducted in the absence of any commercial or financial relationships that could be construed as a potential conflict of interest.
